# Supplementary material for: Patch and matrix characteristics determine the outcome of ecosystem engineering by mole rats in dry grasslands
Source: PeerJ. 2022 Dec 15;10:e14582. doi: 10.7717/peerj.14582 (PMC9760028; doi:10.7717/peerj.14582)
Supplement: Supplemental Information 3 — Value of RRI ranges between −1 and +1. The closer is |RRI| to 1, the higher the contrast between the patches and the matrix. Positive RRI scores indicate that the particular variable has larger values in the mounds, while negative RRI scores indicate that the particular variable has larger values in the matrix grasslands. Lines are fitted for visualising linear relationships. Continuous lines denote significant relationships, while dashed lines denote non-significant relationships. [file peerj-10-14582-s003.pdf]

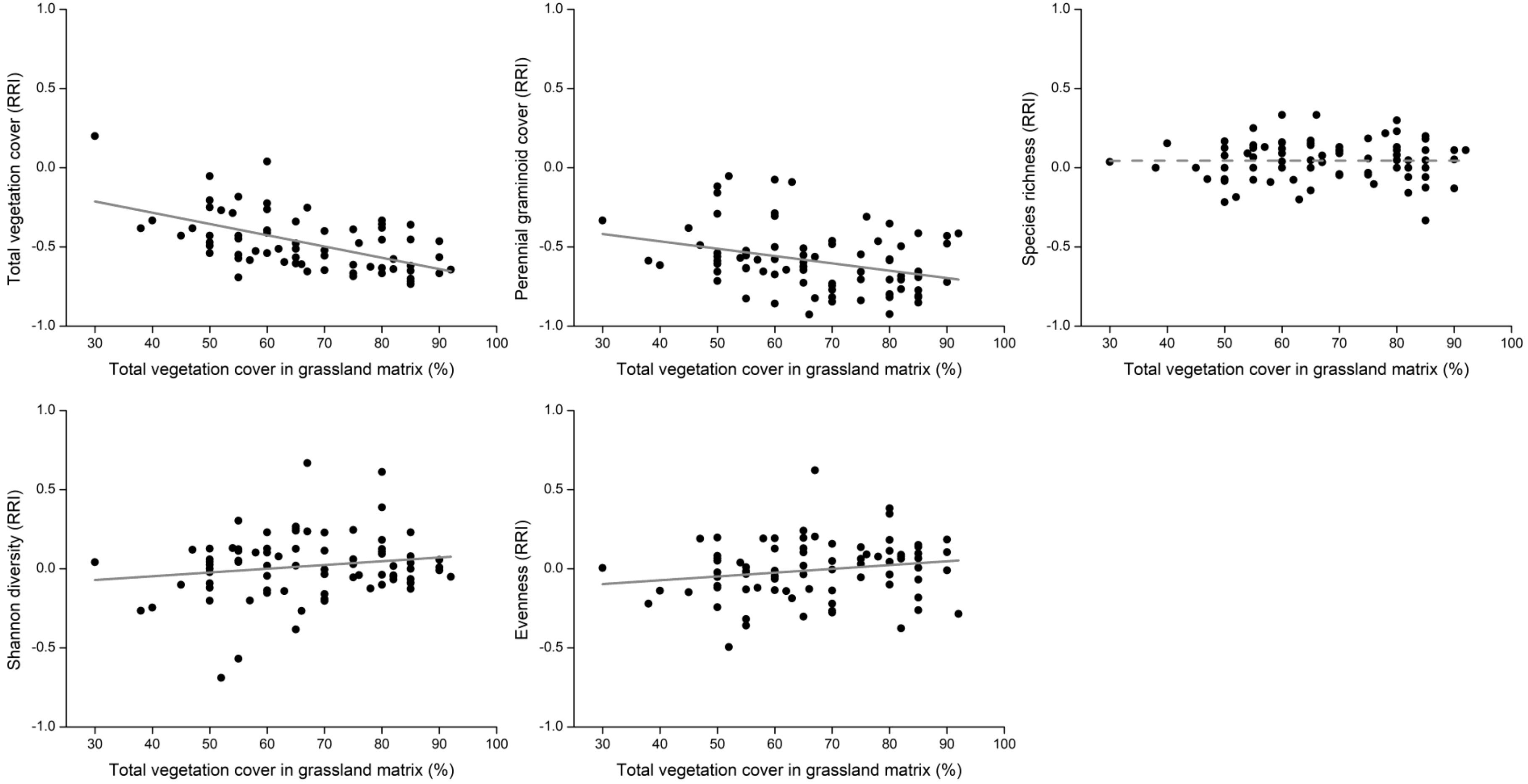

Appendix 3. Relationship between the total vegetation cover in the matrix grasslands and the Relative Response Indexes (RRIs) calculated for the dependent variables (total vegetation cover, perennial graminoid cover, species richness, Shannon diversity and evenness). Value of RRI ranges between −1 and +1. The closer is |RRI| to 1, the higher the contrast between the patches and the matrix. Positive RRI scores indicate that the particular variable has larger values in the mounds, while negative RRI scores indicate that the particular variable has larger values in the matrix grasslands. Lines are fitted for visualising linear relationships. Continuous lines denote significant relationships, while dashed lines denote non-significant relationships.
